# Genetic diversity in India and the inference of Eurasian population expansion

**DOI:** 10.1186/gb-2010-11-11-r113

**Published:** 2010-11-24

**Authors:** Jinchuan Xing, W Scott Watkins, Ya Hu, Chad D Huff, Aniko Sabo, Donna M Muzny, Michael J Bamshad, Richard A Gibbs, Lynn B Jorde, Fuli Yu

**Affiliations:** 1 Department of Human Genetics, Eccles Institute of Human Genetics, University of Utah, 15 North 2030 East, Salt Lake City, UT 84112, USA; 2 Human Genome Sequencing Center, Department of Molecular and Human Genetics, Baylor College of Medicine, One Baylor Plaza, Houston, TX 77030, USA; 3 Department of Pediatrics, University of Washington, 1959 NE Pacific Street, Seattle, WA 98105, USA

## Abstract

**Background:**

Genetic studies of populations from the Indian subcontinent are of great interest because of India's large population size, complex demographic history, and unique social structure. Despite recent large-scale efforts in discovering human genetic variation, India's vast reservoir of genetic diversity remains largely unexplored.

**Results:**

To analyze an unbiased sample of genetic diversity in India and to investigate human migration history in Eurasia, we resequenced one 100-kb ENCODE region in 92 samples collected from three castes and one tribal group from the state of Andhra Pradesh in south India. Analyses of the four Indian populations, along with eight HapMap populations (692 samples), showed that 30% of all SNPs in the south Indian populations are not seen in HapMap populations. Several Indian populations, such as the Yadava, Mala/Madiga, and Irula, have nucleotide diversity levels as high as those of HapMap African populations. Using unbiased allele-frequency spectra, we investigated the expansion of human populations into Eurasia. The divergence time estimates among the major population groups suggest that Eurasian populations in this study diverged from Africans during the same time frame (approximately 90 to 110 thousand years ago). The divergence among different Eurasian populations occurred more than 40,000 years after their divergence with Africans.

**Conclusions:**

Our results show that Indian populations harbor large amounts of genetic variation that have not been surveyed adequately by public SNP discovery efforts. Our data also support a delayed expansion hypothesis in which an ancestral Eurasian founding population remained isolated long after the out-of-Africa diaspora, before expanding throughout Eurasia.

## Background

The Indian subcontinent is currently populated by more than one billion people who belong to thousands of linguistic and ethnic groups [1,2]. Genetic and anthropological studies have shown that the peopling of the subcontinent is characterized by a complex history, with contributions from different ancestral populations [2-5]. Studies of maternal lineages by mitochondrial resequencing have shown that the two major mitochondrial lineages that emerged from Africa (haplogroups M and N, dating to approximately 60 thousand years ago (kya)) are both very diverse among Indian populations [6,7]. Additional studies of mitochondrial haplogroups show that an early migration may have populated the Indian subcontinent, leaving 'relic' populations in present-day India represented by some Austroasiatic-and Dravidian-speaking tribal populations [7-10]. These results highlight that the initial peopling of the Indian subcontinent likely occurred early in the history of anatomically modern humans. Concordant with the mitochondrial DNA (mtDNA) data, paternal lineages within India also show high diversity based on short tandem repeat (STR) markers on the Y chromosome and support an early and continuous presence of populations on the subcontinent [11]. Recent studies of autosomal SNPs and STRs also demonstrate a high degree of genetic differentiation among Indian ethnic and linguistic groups [12-14].

The high diversity and the deep mitochondrial lineages in India support the hypothesis that Eurasia was initially populated by two major out-of-Africa migration routes [3,15-17]. Populations migrating along an early 'southern-route' originated from the Horn of Africa, crossed the mouth of the Red Sea into the Arabian Peninsula, and subsequently migrated into India, Southeast Asia, and Australia. Later, populations migrated out of Africa along a 'northern route' from northern Africa into the Middle East and subsequently populated Eurasia. A recent study suggests that a population ancestral to all Eurasians has limited admixture with Neanderthals after the out-of-Africa migration event but prior to either of the two major Eurasian migrations [18]. This scenario, which we termed the 'delayed expansion' hypothesis [19], predicts that the ancestral Eurasian population separated from African populations long before the expansion into Eurasia. However, the long-term existence of such an ancestral Eurasian population has never been documented. This hypothesis can be tested by using DNA sequence data to examine the demographic history of African populations and a diverse array of Eurasian populations, including previously under-represented samples from South Asia.

Recently, insights into population structure were gained from analyses of data from high-density SNP arrays [13,19-26]. Although high-density SNP genotypes are useful for assessing population structure, quantitative analyses of demographic history depend critically on the patterns of variation represented not just by common SNPs (minor allele frequency ≥0.05) contained in genotyping SNP panels, but also by rare variants (minor allele frequency < 0.05) that have not been thoroughly characterized to date [27]. Furthermore, most SNPs present on the high-density SNP genotyping platforms have been ascertained in an analytically intractable and *ad hoc *fashion [28]. A lack of unbiased polymorphism data limits our ability to accurately estimate the genetic diversity level found in the Indian subcontinent and to correctly infer demographic parameters, such as effective population size, migration rate, and date of population origin and divergence. In addition, despite the large amount of genetic diversity suggested by Y-chromosome, mtDNA, and autosomal microarray analyses, Indian genetic diversity remains largely unexplored by previous large-scale human variant discovery efforts (for example, HapMap and PopRes).

To overcome the limitations and biases associated with SNP microarrays, we used the PCR-Sanger sequencing method to resequence a 100-kb ENCODE region in 92 Indian samples from four population groups (three castes and one tribal population) from the south Indian state Andhra Pradesh and combined our results with eight HapMap populations that are resequenced for the same region [29]. By examining the complete distribution of rare and common variants in several populations that are not included in HapMap/ENCODE studies, we assess the additional information that can be gained by sampling more diverse populations, especially in geographic regions with little or no coverage. Furthermore, using resequencing data from 12 populations covering Africa, Europe, India, and East Asia, we are able to obtain accurate estimates of parameters such as ancestral population sizes and divergence dates and to test the 'delayed expansion' hypothesis of Eurasian population history.

## Results

### ENCODE region selection and SNP discoveries

**Figure 1:**
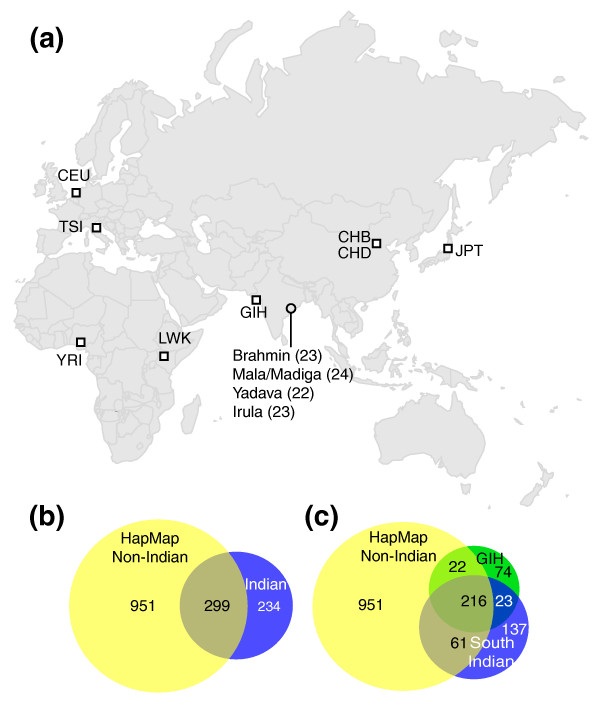
**SNP discovery in Indian populations**. (a) Population samples. The number of individuals sampled from each Indian population is shown. **(b) **The number of SNPs found in HapMap non-Indian and Indian populations. **(c) **The number of SNPs found in south Indian, HapMap GIH, and HapMap non-Indian populations. HapMap non-Indian populations include CEU, CHB, CHD, JPT, LWK, TSI, and YRI. South Indian populations include Brahmin, Irula, Mala/Madiga, and Yadava.

We sequenced one 100-kb ENCODE region-ENr123 (hg18: Chr12 38,826,477-38,926,476) in four different Andhra Pradesh ethnic groups representing three castes, Brahmin, Yadava, and Mala/Madiga, and one tribal group Irula (Figure 1a). We chose ENr123 because it has a low gene density and should represent a selectively neutral region (gene density of 3.1% and non-exonic conservation rate of 1.7%). Among the 92 individuals that passed quality-control steps, a total of 453 SNPs were identified, corresponding to a SNP density of one SNP per 221 bp. To determine the accuracy of the newly identified SNPs, we carried out additional experiments using the Roche 454 sequencing platform to validate the Indian-specific SNPs in individuals with heterozygous genotypes (see Materials and methods for details). The validation results showed that the genotypes of new SNPs have a high confirmation rate (approximately 80% for heterozygous SNPs). For alleles that have been seen only once in the dataset, the confirmation rate is greater than 85% (Supplemental Table S1 in Additional file 1).

To generate a comparable dataset, we applied the same SNP calling criteria on 722 HapMap individuals who were sequenced using the same protocol in the ENCODE3 project [29]. We then merged these two datasets (four Indian populations and eight HapMap populations (CEU, CHB, CHD, GIH, JPT, LWK, TSI, and YRI)) to obtain a final data set that consists of 1,484 SNPs in 722 individuals from 12 populations (see Materials and methods for SNP merging and filtering details).

Among the 1,484 total SNPs, 234 (15.8%) are specific to Indian populations (four Andhra Pradesh populations and the HapMap northern Indian GIH; Figure 1b). For Indian individuals, the average number of specific SNPs per individual is 1.5. This number is lower than in HapMap African individuals (2.4 SNPs), but higher than both HapMap European (1.3 SNPs) and HapMap East Asian individuals (1.1 SNPs). This result suggests that higher autosomal genetic diversity is harbored in Indian samples compared to other HapMap Eurasian samples. Among the 453 SNPs in the four newly sequenced south Indian populations, 137 (30%) are not present in any HapMap populations (Figure 1c), including one novel non-synonymous singleton variant (Supplemental text in Additional file 1).

### Genetic diversity in India

Because many genetic diversity measurements are influenced by sample size, we normalized the sample size of each group by randomly selecting a subset of HapMap individuals to match the sample size of the Indians. For convenience, we denote four groups of populations (African, East Asian, European, and Indian) as 'continental groups'. For continental groups, 152 unrelated individuals were randomly selected from HapMap African, European, and East Asian samples, respectively (matching the 152 Indian individuals in the dataset). At the population level, 24 individuals were randomly selected from each HapMap population, and all individuals from south Indian populations were included in the analyses. After sample size normalization, we measured genetic diversity using various summary statistics, including the number of segregating sites (*S*), Watterson's *θ *estimator, nucleotide diversity (*π*), and observed SNP heterozygosity (*H*) for each population and continental group (Table 1). We also evaluated the haplotype diversity in each group by averaging the haplotype heterozygosity in ten 10-kb non-overlapping windows and tested the neutrality of the region using the Tajima's *D *test. The Tajima's *D *test result was consistent with neutrality, providing no evidence for either positive or balancing selection in this region (Table 1), as expected given the low gene density in this region.

**Table 1:** Genetic diversity in continental groups and populations

	*nInd*	*S*	*Sp*	*θ*	*π *(π10^-5^)	*H *(π10^-5^)	Hap Het	Tajima's D	*P*
Continent									
India	152	533	237	84.70 (82.72-86.68)	83.68 (79.20-88.17)	77.53	0.89	-0.04	0.97
Africa	152	656	416	104.25 (101.82-106.68)	85.28 (80.71-89.86)	78.03	0.95	-0.57	0.57
Europe	152	535	205	85.02 (83.03-87.01)	74.64 (70.63-78.65)	67.95	0.88	-0.38	0.70
East Asia	152	436	186	69.29 (67.66-70.92)	73.61 (69.66-77.57)	73.10	0.90	0.19	0.85
Population									
Brahmin	23	287	16	65.30 (59.72-70.88)	75.08 (64.51-85.64)	60.02	0.79	0.55	0.58
GIH	24	282	47	63.54 (58.27-68.81)	72.41 (62.45-82.38)	60.96	0.87	0.51	0.61
Irula	23	292	20	66.44 (60.76-72.12)	82.77 (71.13-94.40)	95.12	0.89	0.90	0.37
Mala/Madiga	24	342	46	77.06 (70.69-83.43)	84.46 (72.85-96.07)	89.33	0.87	0.35	0.73
Yadava	22	317	28	72.87 (66.45-79.29)	88.94 (76.15-101.73)	92.82	0.95	0.81	0.42
LWK	24	359	85	80.89 (74.21-87.57)	82.51 (71.17-93.86)	85.81	0.96	0.07	0.94
YRI	24	349	91	78.64 (72.14-85.14)	82.03 (70.75-93.31)	76.86	0.95	0.16	0.88
CEU	24	262	43	59.04 (54.13-63.94)	70.64 (60.91-80.37)	77.68	0.85	0.72	0.47
TSI	24	298	58	67.15 (61.58-72.71)	73.95 (63.78-84.13)	72.54	0.89	0.37	0.71
CHB	24	254	34	57.23 (52.47-61.99)	76.49 (65.97-87.01)	78.88	0.90	1.23	0.22
CHD	24	212	24	47.77 (43.78-51.76)	69.87 (60.24-79.49)	72.34	0.81	1.68	0.09
JPT	24	236	34	53.18 (48.75-57.61)	73.66 (63.52-83.80)	62.88	0.88	1.40	0.16

*nInd*, number of individuals; *S*, number of segregating sites; *Sp*, number of private segregating sites; *θ*, estimated theta (4N_e_u) from *S*; *π*, nucleotide diversity; *H*, observed heterozygosity; Hap Het, averaged haplotype diversity over ten 10-kb windows; Tajima's *D*, Tajima's *D*; *P*, *P*-value for Tajima's *D *test. Confidence intervals of *θ *and *π *are shown in parentheses.

At the population level, *π *and *H *indicate that some Indian populations have diversity levels comparable to or even higher than those of HapMap African populations. Specifically, Mala/Madiga, Yadava, and Irula have the highest *π *among all populations (84.46 π 10^-5^, 88.94 π 10^-5^, and 82.77 π 10^-5^, respectively). In contrast, Brahmins and HapMap GIH have lower diversity levels, comparable to HapMap European and East Asian populations (Table 1). Due to small sample sizes, the confidence intervals of *π *for all populations overlap. However, at the continental level, Indians have significantly higher nucleotide diversity than Europeans and East Asians, although *θ *and haplotype diversity are similar among the three groups (Table 1). Removal of unconfirmed genotypes in Indian individuals does not change the results (Supplemental text and Supplemental Table S3 in Additional file 1).

**Figure 2:**
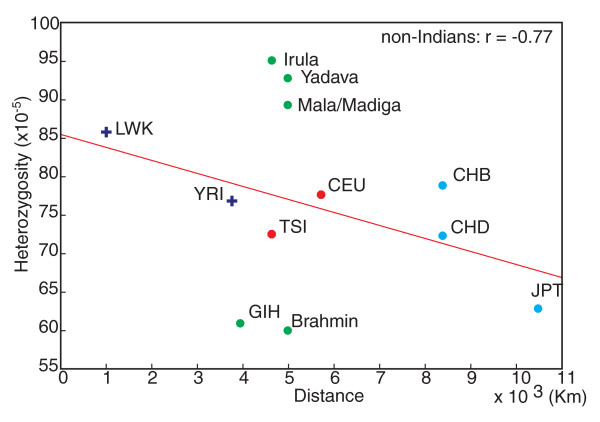
**Population SNP heterozygosity as a function of geographic distance from eastern Africa**. The correlation coefficient of HapMap non-Indian populations is shown.

Several studies have shown that heterozygosity decreases with increasing distance from eastern Africa, presumably due to multiple bottlenecks that human populations experienced during the migration [22,30]. Among non-Indian populations, we observed a significant negative correlation between *H *and the distance to eastern Africa (Figure 2; r = -0.77, *P *= 0.04). However, when the Indian populations were included, the correlation became non-significant (r = -0.33, *P *= 0.29). This lack of correlation is due to large variation in *H *among the Indian populations (60.02 π 10^-5 ^in Brahmins to 95.12 π 10^-5 ^in the Irula). This result demonstrates great variation in diversity among groups within India.

### Demographic history of Eurasian populations

**Figure 3:**
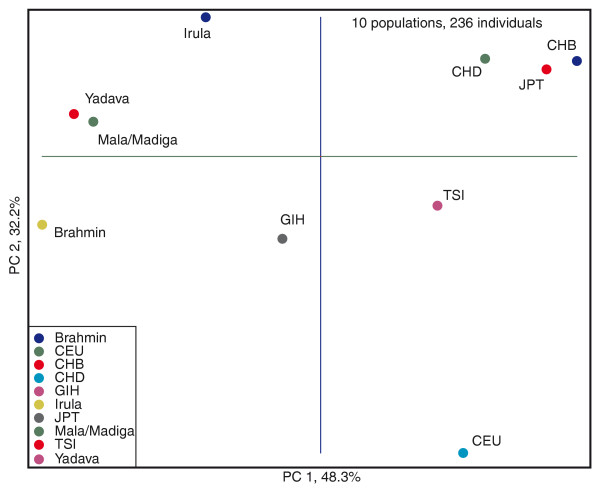
**Principal components analysis of Eurasian populations**. The first two principal components (PCs) and the percentage of variance explained by each PC are shown.

To study the relationship among populations, we first performed principal components analysis (PCA) on the genetic distances between populations using the normalized dataset. When all populations are included in the analysis, the first principal component (PC1) accounts for 93% of the total variance and separates African and non-African populations (Supplemental Figure S1 in Additional file 1). In PCA of only Eurasian populations, PC1 separates Indian populations from European and East Asian populations, and PC2 separates European and Asian populations (Figure 3). Among Indian populations, the tribal Irula and HapMap GIH have the shortest distance to East Asian populations while Brahmin has the largest distance. The northern Indian GIH population diverges from south Indians and its closest relationship is with HapMap TSI populations. This observation is consistent with the general genetic cline in India observed in previous studies [13,31]. We also performed PCA and *ADMIXTURE *analysis at the individual level (Supplemental Figure S2 in Additional file 1). Because of the relatively small size of our dataset, individuals are not tightly clustered as seen in studies with genome-wide data [19,22,23]. The African individuals are separated from the Eurasian individuals, but Eurasian individuals from different populations are not separated into distinct clusters.

Next, we examined the divergence between Indian and non-Indian populations using pairwise *F_ST _*estimates. In comparing major continental groups, India and Europe have the smallest *F_ST _*value (Table 2). At the individual population level, however, Indian populations show varying affinities to other Eurasian populations: the Indian tribal population (Irula) shows closer affinity to HapMap East Asian populations while the HapMap GIH and the Brahmin show a closer relationship to HapMap European populations. The Mala/Madiga and Yadava show a similar distance to the HapMap European and East Asian populations (Table 3). Among Indian populations (Supplemental Table S2 in Additional file 1), the smallest *F_ST _*value is between Yadava and Mala/Madiga (0.1%), and the largest *F_ST _*value is between HapMap GIH and the tribal Irula (10.4%).

**Table 2:** Pairwise *F_ST_*values (%) between and among continental groups

	Africa	Europe	India	East Asia
Africa	12.7			
Europe	28.9	8.2		
India	30.3	6.1	6.7	
East Asia	31.5	10.9	7.8	3.3

The within continent (among populations) *F_ST _*values are shown on the diagonal line.

**Table 3:** Pairwise *F_ST_*values (%) between Indian and HapMap non-Indian populations

	LWK	YRI	CEU	TSI	CHB	CHD	JPT
Brahmin	35.1	37.6	12.3	9.5	18.0	13.0	17.0
GIH	32.6	34.9	11.5	6.2	11.5	5.9	10.0
Mala/Madiga	31.7	34.3	10.4	6.7	12.8	8.1	11.8
Yadava	31.8	34.5	12.8	9.1	12.9	8.9	12.2
Irula	33.2	35.4	15.8	11.5	8.3	6.2	8.0

The complete sequence data allow us to obtain an accurate derived-allele frequency (DAF) spectrum. At both the continental and population levels, the DAF spectra in our dataset are characterized by a high proportion of low-frequency SNPs, as expected for sequencing data (Supplemental text and Supplemental Figure S3 in Additional file 1). Based on the DAF spectra, we are able to infer the parameters associated with Indian population history, such as the divergence time, effective size, and migration rate between populations using the program *∂a∂i *(*Diffusion Approximation for Demographic Inference*) [32].

**Figure 4:**
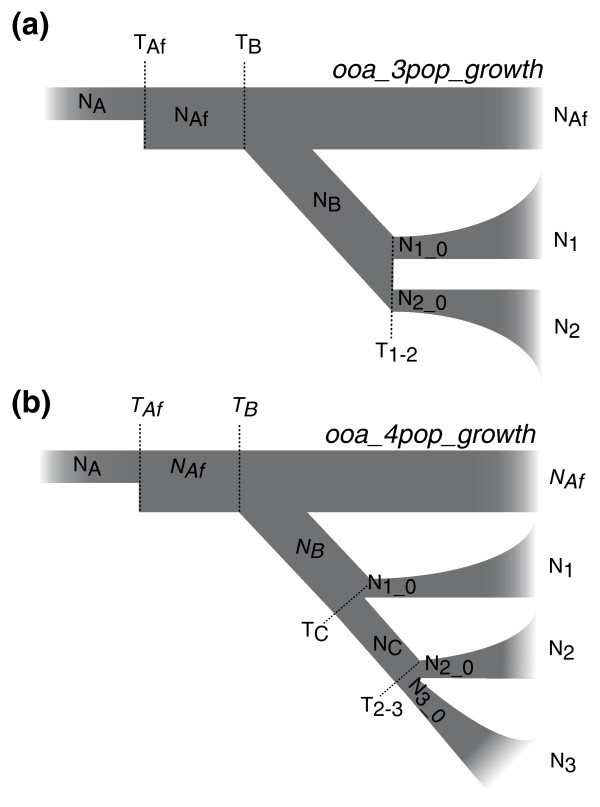
**Illustration of the ***∂a∂i ***models**. (a) Three-population out-of-Africa model. The ten parameters estimated in the model (*N_A_*, *N_Af_*, *N_B_*, *N_1_0_*, *N_1_*, *N_2_0_*, *N_2_*, *T_Af_*, *T_B_*, *T_1-2_*,) are shown. **(b) **Four-population out-of-Africa model. The ten parameters estimated in the model (*N_A_*, *N_C_*, *N_1_0_*, *N_1_*, *N_2_0_*, *N_2_*, *N_3_0_*, *N_3_*, *T_C_*, *T_2-3_*,) are shown. *N_Af_*, *N_B_*, *T_Af_*, and *T_B _*are fixed in this model.

Because *∂a∂i *can simultaneously infer population parameters in models involving three populations, we first estimated the parameters associated with the out-of-Africa event using the African continental group and two continental Eurasian groups. We started from a simplified three-population divergence model based on the out-of-Africa model described in *∂a∂i *[32] and assessed the model-fitting improvement of adding different parameters to the model (Supplemental text in Additional file 1). Our results suggest that allowing exponential growth in the Eurasian continental groups substantially improves the model. On the other hand, allowing migrations among groups provides little improvement in the data-model fitting, suggesting that little gene flow occurred between the continental groups (Supplemental Figure S5 in Additional file 1). Therefore, we inferred the parameters from the three-population out-of-Africa model, allowing exponential growth in the Eurasian groups but no migration among groups (Figure 4a). Under this model, a one-time change in African population size occurs at time *T_Af _*before any population divergence, and the population size changes from the ancestral population size *N_A _*to *N_Af _*in Africa. At time *T_B _*the Eurasian ancestral population with a population size of *N_B_* diverges from the African population, while the African population size *N_Af _*remains constant until the present. The two Eurasian groups split from the ancestral population *N_B _*at time *T_1-2_*, with initial population sizes of *N_1_0 _*and *N_2_0_*, respectively. Both populations experience exponential population size changes from the time of divergence to reach the current population sizes *N_1 _*and *N_2_*.

The inferred parameters between continental groups, along with confidence intervals (CIs) for each parameter, are shown in Table 4. When the mutation rate is set at 1.48 π 10^-8 ^per base pair per generation (see Materials and methods for mutation rate estimate), the ancestral population size is estimated to be between 13,000 and 14,000 for all models (Table 4). The African effective population size estimates (*N_Af_*, 18,036 to 18,976; CI, 15,077 to 22,673) are comparable to the size of the Eurasian ancestral population (*N_B_*, 12,624 to 21,371; CI, 7,360 to 32,843). At the time of the Eurasian population divergence, the population sizes of the two Eurasian continental groups in each model (*N_1_0 _*and *N_2_0_*) are consistently smaller than the African and the Eurasian ancestral population sizes, with one exception for the estimated European population size (25,543; CI 6,101 to 29,016) in the Africa-East Asia-Europe model. These results suggest that the Eurasian population experienced population bottlenecks at the time of their divergence. Among Eurasians, East Asians have the smallest effective population size at the time of divergence (approximately 1,500; CI, 779 to 3,703; Table 4). The divergence time estimates between Africans and non-Africans range from 88.4 to 111.5 kya and the CIs of all three estimates overlapped, consistent with the existence of a single ancestral Eurasian population. The three non-African continental groups diverged from each other more recently than 40 kya: East Asians were separated from Indians (39.3 kya; CI, 29.7 to 59.1) and Europeans (39.2 kya; CI, 29.8 to 55.8) before the divergence of Indians and Europeans (26.6 kya; CI, 20.1 to 40.8). Overall, these results support a scenario in which the ancestors of the Indian, European, and East Asian individuals left Africa in one major migration event, and then diverged from one another more than 40,000 years later.

**Table 4:** *∂a∂i*inferred parameters for the three-population out-of-Africa model

Continent 1	Africa	Africa	Africa
Continent 2	East Asia	India	India
Continent 3	Europe	East Asia	Europe
*N_A_*	13,107	13,647	13,390
*N_Af_*	18,976 (15,077-22,673)	18,036 (15,277-20,401)	18,387 (14,948-20,674)
*N_B_*	12,624 (7,360-21,768)	18,923 (8,230-32,825)	21,371 (13,078-31,684)
*N_1_0_*	1,563 (903-2,760)	4,073 (1,791-27,445)	1,829 (1,055-5,463)
*N_1_*	40,488 (20,734-77,945)	36,425 (11,976-86,661)	75,961 (20,902-137,972)
*N_2_0_*	25,543 (6,101-29,016)	1,504 (780-3,702)	3,471 (1,813-25,273)
*N_2_*	18,400 (14,733-52,112)	39,580 (18,835-91,179)	70,960 (17,890-139,643)
*T_Af _*(kya)	115.4 (62.9-219.7)	112.0 (72.0-728.2)	113.7 (77.0-411.0)
*T_B _*(kya)	88.4 (62.5-125.4)	111.5 (72.0-150.2)	103.9 (76.8-134.5)
*T_1-2 _* (kya)	39.2 (29.8-55.8)	39.3 (29.7-59.1)	26.6 (20.8-40.8)
Maximum likelihood	-1,232.6	-1,272.6	-1,276.0

Confidence intervals are shown in parentheses.

To further examine the population history among Eurasian populations, we constructed a four-population model containing all four continental groups (Figure 4b). Because parameters from only three populations can be estimated by *∂a∂i *at the same time, we fixed the parameters of the out-of-Africa epoch (*N_Af _*, *N_B_*, *T_Af_*, and *T_B_*) in the model based on the parameters estimated from the three-population model with the highest likelihood (Africa-East Asia-European), as described in *∂a∂i *[32]. A model comparison again suggests that adding migrations to the model does not substantially improve the model-fitting (Supplemental text and Supplemental Figure S6 in Additional file 1). Therefore, migrations were excluded from the model to reduce the number of inferred parameters and to improve the speed of computation. Among the three population divergence scenarios, two models ('East Asia first' and 'India first') showed similar maximum likelihood values (-1,278.9 and -1,278.7, respectively), indicating comparable fitting to the data. In contrast, the 'Europe first' model has a substantially lower maximum likelihood value (-1,280.7), suggesting that this model is less plausible. The estimated parameters for the 'East Asia first' and the 'India first' models are shown in Table 5. Consistent with the three-population models, the 'East Asia first' mode estimates that East Asians diverged from the ancestral Eurasian population approximately 44 kya, and Europeans and Indians diverged approximately 24 kya. Interestingly, the 'India first' model suggests that the divergence time among the three continental groups are similar, with Indians diverging only 0.2 kya before Europeans and East Asians. Under this model, the initial population size of the Indian population (*N_1_0_*, 11,410; CI, 4,568 to 28,665) is comparable to the Eurasian ancestral population size (*N_B_*, 12,345), consistent with the high diversity we observed in these Indian samples.

**Table 5:** *∂a∂i*inferred parameters for the four-population out-of-Africa model

	Model	
	
	East Asia first	India first
		
Continent 1	East Asia	India
Continent 2	India	East Asia
Continent 3	Europe	Europe
*N_A_*	13,195	13,483
*N_Af_*^a^	19,023	19,438
*N_B_*^a^	12,081	12,345
*N_1_0_*	2,003 (1,198-3,529)	11,410 (4,568-28,665)
*N_1_*	31,020 (16,773-54,561)	18,182 (9,643-45,162)
*N_C_*	77,786 (25,900-143,596)	171 (14-127,200)
*N_2_0_*	1,881 (1,160-5,214)	1,735 (891-2,571)
*N_2_*	77,285 (21,282-135,595)	33,571 (20,084-66,737)
*N_3_0_*	2,029 (1,552-6,314)	11,689 (3,309-27,864)
*N_3_*	131,889 (26,976-142,541)	28,370(15,869-64,163)
*T_Af _*(kya)^a^	119.6	117.8
*T_B _*(kya)^a^	92.2	89.8
*T_C _*(kya)	43.9 (25.9-69.3)	40.5 (30.9-56.2)
*T_2-3 _*(kya)	23.9 (18.2-35.6)	40.3 (31.0-44.6)
Maximum likelihood	-1,278.9	-1,278.7

^a^*N_Af _*, *N_B_*, *T_Af_*, and *T_B _*were fixed in the model based on the best parameters from the three-population model. Confidence intervals are shown in parentheses.

When individual populations are analyzed, the patterns are largely consistent with the results from continental groups (Supplemental text and Supplemental Table S4 in Additional file 1). The CIs around the parameters are generally larger, indicating a loss of power due to the smaller sample sizes of the individual populations compared to the continental groups.

## Discussion

India has served as a major passageway for the dispersal of modern humans, and Indian demographics have been influenced by multiple waves of human migrations [3,9,33]. Because of its long history of human settlement and its enormous social, linguistic, and cultural diversity, the population history of India has long intrigued anthropologists and human geneticists [3,12-14,20,34,35]. A better understanding of Indian genetic diversity and population history can provide new insights into early migration patterns that may have influenced the evolution of modern humans.

By sampling and resequencing 92 south Indian individuals we found 137 novel SNPs in the 100-kb region. These new SNPs represent approximately 30% of the total SNPs in these individuals. This result is consistent with several previous studies that showed that genetic variants in Indian populations, especially the less common variants, are incompletely captured by HapMap populations [12,29,36]. More importantly, we found that genetic diversity varies substantially among Indian populations. At the continental level, the Indian continental group has significantly higher nucleotide diversity than both European and East Asian groups. Although the HapMap GIH and the Brahmin populations have genetic diversity values comparable to those of other HapMap Eurasian populations, diversity values (*π *and *H*) in the Irula, Mala/Madiga, and Yadava samples are higher than those of the HapMap African populations. The genetic diversity difference among Indian populations has been observed previously in mitochondria [37], autosomal [34], and Y chromosome [11] studies. Even among geographically proximate populations, genetic diversity can vary greatly due to differences in effective population sizes, mating patterns, and population history among these populations. Our finding highlights the importance of including multiple Indian populations in the human genetic diversity discovery effort.

**Figure 5:**
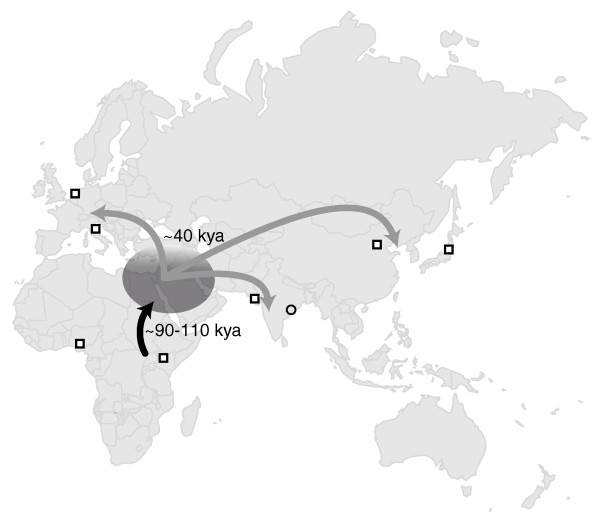
**The 'delayed expansion' hypothesis**. In this hypothesis, the ancestal Eurasian population separated from African populations approximately 100 kya but did not expand into most of Eurasia until approximately 40 kya.

Because sequence data are free of ascertainment bias, we were able to study the relationship between populations in detail. In addition to examining population differentiation (by *F_ST _*estimates) and population structure, we inferred the divergence time and migration rate among continental groups using the program *∂a∂i*. The estimates of continental *F_ST _*values and PCA results show that the greatest population differentiation occurs between African and non-African groups, while the least amount of differentiation occurs between Europeans and Indian populations. This is consistent with the estimates of divergence time between continental groups based on the three-population models (Table 4): the divergence time between African and the ancestral Eurasian population (88 to 112 kya; CI, 63 to 150 kya) is much older than the divergence time among the Eurasian groups (27 to 39 kya; CI, 20 to 59 kya). The more recent divergence time and the low migration rate estimates among the current Eurasian populations support the 'delayed expansion' hypothesis for the human colonization of Eurasia (Figure 5). Consistent with previous studies [18,19], these estimates indicate that a single Eurasian ancestral population remained separated from African populations for more than 40,000 years prior to the population expansion throughout Eurasia and the divergence of individual Eurasian populations.

Although this Eurasian ancestral population would have been isolated from the sub-Saharan African populations in this study, the geographic location of this population is uncertain. The most plausible location is the Middle East and/or northern Africa. A Middle East location of this population could explain the admixture patterns of Neanderthal and the non-African populations [18], although current archeological evidence does not support continuous occupation of the Middle East by modern humans prior to the Eurasian expansion [38]. Alternatively, a north African location is more consistent with the archeological record but requires extreme population stratification within Africa [39]. A more comprehensive sampling of African populations could help to pinpoint the location of this population.

Under the four-population out-of-Africa model, the divergence times among the three Eurasian continental groups are similar. The likelihood of the model with an earlier East Asian divergence is similar to that of the model with an earlier Indian divergence. This result appears to contradict the hypothesis that the Indian sub-continent was first populated by an early 'southern-route' migration through the Arabian Peninsula [3,15-17]. Previous studies have identified unique mitochondrial M haplogroups in some tribal populations that are consistent with an older wave of migration [7-9]. For example, some Dravidian-and Austroasiatic-speaking Indian tribal populations share ancestral markers with Australian Aborigines on a mitochondrial M haplogroup (M42), which is dated to approximately 55 kya [40]. However, because our samples of the Indian continental group are composed of three caste populations and one tribal Indian population, these populations are unlikely to effectively represent the descendants of the early 'southern-route' migration event. This sample collection might partially explain why we were unable to distinguish the 'East Asia first' model from the 'India first' model.

The between-population *F_ST _*estimates and divergence time estimates show that the Indian populations have different affinities to European and East Asian populations. South Indian Brahmin and northern Indian GIH have higher affinity to Europeans than to East Asians, while the tribal Irula generally have closer affinity to East Asian populations. The differential population affinities of Indian populations to other Eurasian populations have been observed previously using mtDNA, Y-chromosome, and autosomal markers. Regardless of caste affiliation, genetic distance estimates with mitochondrial markers showed a greater affinity of south Indian castes to East Asians, while distance estimates with Y-chromosome markers showed greater affinity of Indian castes to Europeans [14,41,42]. Distances estimated from autosomal STRs and SNPs also showed differential affinity of caste populations to European and East Asian populations [12-14,20].

There are some limitations on our ability to infer demographic history in this study. First, our results are based on the sequence of a continuous 100-kb region. Therefore, these results reflect the history of a number of possibly co-segregating markers from a small portion of the genome. Our CIs around the parameter estimates, however, account for this co-segregation. Second, although we incorporated a number of parameters of population history, our demographic model is still a simplification of the true population history. Third, parameters estimated in our model are dependent on the estimate of the human mutation rate, which varies several-fold using different methods or datasets [43,44]. Nevertheless, with appropriate caution, the sequence data allow us to explore demographic models in ways that are not possible with genotype data alone.

## Conclusions

By sequencing a 100-kb autosomal region, we show that Indian populations harbor large amounts of genetic variation that have not been surveyed adequately by public SNP discovery efforts. In addition, our results strongly support the existence of an ancestral Eurasian population that remained separated from African populations for a long period of time before a major population expansion throughout Eurasia. With the rapid development of sequencing technologies, in the near future we will obtain exome and whole-genome data sets from many diverse populations, such as isolated Indian tribal groups who might better represent the descendants of a 'southern-route' migration event. These data will allow us to evaluate more complex models and refine the demographic history of the human Eurasian expansion.

## Materials and methods

### DNA samples, DNA sequencing and SNP calling

Ninety-four individuals from three caste groups and one tribal group from Andhra Pradesh, India were sampled (Figure 1a). All samples belong to the Dravidian language family and were collected as unrelated individuals as described previously [45,46]. All studies of South Indian populations were performed with approval of the Institutional Review Board of the University of Utah and Andhra University, India. To sequence the ENCODE region ENr123, we used the same sets of primers that were used for the ENCODE3 project for PCR amplification and the same Sanger sequencing. Next, we obtained the sequence of 722 HapMap individuals from the ENCODE3 project [29] and performed SNP calling using the same SNP discovery pipeline [47]. This experimental design allowed us to directly compare genetic variation patterns observed in these Indian populations with those observed in the HapMap populations studied by ENCODE3 [29]. The sequence traces of the Indian samples generated from this study can be accessed at NCBI trace archive [48] by submitting the query: center_project = 'RHIDZ'.

### SNPs and individual selection

After the SNP-calling process, two individuals with less than 80% call rates were removed from the dataset (one Brahmin and one Yadava). The SNP calls from the remaining 92 samples that passed quality control were then combined with the SNP calls from eight HapMap non-admixed populations studied by ENCODE3, including individuals from the Centre d'Etude du Polymorphisme Humain collection in Utah, USA, with ancestry from Northern and Western Europe (CEU), Han Chinese in Beijing, China (CHB), Japanese in Tokyo, Japan (JPT), Yoruba in Ibadan, Nigeria (YRI), Chinese in Metropolitan Denver, CO, USA (CHD), Gujarati Indians in Houston, TX, USA (GIH), Luhya in Webuye, Kenya (LWK), and Toscani in Italy (TSI), to create a final dataset containing 722 individuals from 12 populations.

After merging the HapMap and the south Indian data sets, 112 loci that are fixed in all 12 populations were removed from the dataset. Thirteen tri-allelic SNPs were also removed because most analyses in this study are designed for bi-allelic SNPs. For SNPs that are fixed in certain populations, genotypes were filled-in using the hg18 reference allele because the reference allele information was used in the SNP calling process (that is, only genotypes that are different from the reference alleles are called as SNPs).

The Hardy-Weinberg equilibrium test was performed on each of the 12 populations, and *P*-values from each test were obtained and transformed to Z-scores. Twelve Z-scores were combined to a single Z-score and transformed to a single *P*-value for each SNP. Bonferroni correction was used, and 48 SNPs that failed the test at the 0.01 level (*P* < 0.01/1,532) were removed. The ancestral/derived allele states of each SNP were determined using the human/chimpanzee alignment obtained from the UCSC database (hg18 vs.panTro2 [49]). Minor-alleles of 17 SNPs were assigned as the derived allele because the derived allele could not be determined by human-chimpanzee alignments. Genotypes of all samples in the final dataset are available as a supplemental file on our website [50] under Published Data.

### SNP validation

For the 137 SNPs that are specific to our samples (that is, not present in any HapMap populations), we performed a validation experiment using an independent platform (Roche 454). When the minor allele is present in more than five individuals at a given locus, five individuals with the heterozygous genotype were randomly selected for validation. Among the 137 SNPs, we successfully designed and assayed 119 SNPs in 211 individual experiments. For the validation pipeline, we used PCR to amplify regions around the variants using the same primers as those used in the initial variant detection pipeline. In order to make genotype calls on all experiments simultaneously and also to reduce the cost of Roche 454 sequencing, we pooled PCR reactions in ten different pools and each pool was sequenced using a quarter of a Roche Titanium 454 sequencing run. The analysis was done using the Atlas-SNP2 pipeline available at the BCM-HGSC [51]. Reads from the 454 runs were anchored using BLAT [52] to a unique spot in the genome, followed with the refined alignments using the cross_match program [53]. We required at least 50 reads mapped to the variant site to make a validation call and the fraction of reads with the variant to be >15% of all reads mapping to that site.

### Sequence statistics, F_ST _estimates, and PCA

Sequence-analysis statistics (*S*, *θ*, *π*, *H *and Tajima's *D*), and the confidence intervals for *θ *and *π *were calculated using the Population Genetics and Evolution Toolbox [54] in MATLAB (version r2009a). To assess haplotype diversity, the dataset was phased using fastPHASE (version 1.2) [55] with imputation, and the phased dataset was separated into ten 10-kb non-overlapping windows. Haplotype heterozygosity was then calculated for each window, and the mean heterozygosity for each population/continental group was calculated. For the SNP heterozygosity/geographic distance correlation analysis, the great-circle distance between each population and Addis Ababa, Ethiopia, a proposed point of modern human origin [56], was calculated. For populations that were collected from places other than their origins, an approximate origin location was used, such as Beijing, China for CHD, and Gujarat, India for GIH. *F_ST _*estimates between populations were calculated by the method described by Weir and Cockerham [57]. Nei's genetic distances between populations were estimated from allele-frequency data as implemented in the PHYLIP software package [58] and PCA was performed using MATLAB.

### Demographic history inference

Demographic history parameters were inferred using the program *∂a∂i *(version 1.5.2) [59]. Using a diffusion approximation to the allele-frequency spectrum, *∂a∂i *implements a series of methods to infer population history based on sequence data. We compared three different three-population out-of-Africa models and three four-population out-of Africa models to test the effect of adding different parameters to the model (Supplemental text and Supplemental Figures S5 and S6 in Additional file 1). For the two models used in the final analysis, the python programs that were used to estimate the parameters, including the function calls, grid sizes, initial parameters, and parameter boundaries, are shown in Supplemental Figures S7 and S8 in Additional file 1. To ensure that the algorithm identified the optimal parameters, ten independent runs were performed on each model, and the parameter set with the highest likelihood was selected as the final result. For each model, 500 bootstrap replicates were performed on the dataset to obtain the confidence intervals. The per-generation mutation rate was estimated based on the human-chimpanzee divergence in this region (1.2%) using the method described in [43], with a generation time of 25 years, a human-chimpanzee speciation time of 6 million years ago, and a human-chimpanzee ancestral effective population size of 84,000 (averaged from the estimates from [60-62]).

## Abbreviations

Bp: base pair; CI: confidence interval; DAF: derived-allele frequency; kb, kilo-base; kya, thousand years ago; PC: principal component; PCA: principal components analysis; SNP: single nucleotide polymorphism; STR: short tandem repeat.

## Competing interests

The authors declare that they have no competing interests.

## Authors' contributions

JX, LJ, and FY conceived and designed the study. JX, WSW, YH, CH, and FY performed the analysis and wrote the manuscript. AS and DM generated sequencing data and performed the validation experiment. MB collected the Indian samples. RG, LJ, and FY participated in project coordination. All authors read and approved the final manuscript.

## Supplementary Material

Additional file 1 **Supplemental text, four supplemental tables, and nine supplemental figures**. Click here for file
