# Supplementary material for: Genetic diversity in India and the inference of Eurasian population expansion
Source: Genome Biol. 2010 Nov 24;11(11):R113. doi: 10.1186/gb-2010-11-11-r113 (PMC3156952; doi:10.1186/gb-2010-11-11-r113)
Supplement: Additional file 1 — Supplemental text, four supplemental tables, and nine supplemental figures. [file gb-2010-11-11-r113-S1.pdf]

Supporting Online Material for

Genetic diversity in India and the inference of Eurasian population expansion

Jinchuan Xing, W. Scott Watkins, Ya Hu, Chad D. Huff, Aniko Sabo, Donna M. Muzny, Michael J. Bamshad, Richard A. Gibbs, Lynn B. Jorde, and Fuli Yu

Supplemental Text

Indian-specific coding variants

Because this region contains seven exons of the *LRRK2* gene (leucine-rich repeat kinase 2, RefSeq id: NM_198578), we determined if any of the Indian-specific SNPs reside in the coding region of the gene. Among a total of ten coding SNPs in the whole dataset (three synonymous and seven non-synonymous mutations), one SNP that is within exon six of the *LRRK2* gene is specific to Indians. This novel T -> C mutation (hg18 chr12:38,920,671) causes a non-synonymous change at the amino acid 231 of the protein and changes the serine (TCC) to a proline (CCC). This mutation is present as a heterozygote in one Yadava individual, and it is predicted to be "possibly damaging" by PolyPhen [1]. The *LRKK2* gene belongs to the leucine-rich repeat kinase family and encodes a protein called dardarin. Mutations in this gene are known to cause Parkinson disease [2].

Indian genetic diversity after removing un-confirmed SNPs

To confirm the validity of our results, we replaced the 42 genotypes that are discordant between the initial experiments and the validation experiments with genotypes from the 454 sequencing experiment and re-analysis the data. The removal of un-confirmed genotypes eliminates19 SNP loci in Indian individuals but does not change the results and conclusions based on the initial data. The Indian continental group still has significantly higher π than European and East Asian groups. The highest π and H are still observed in Indian populations (Supplemental Table 3).

Derived-allele frequency (DAF) spectra

Using the normalized datasets, we determined the DAF spectrum in each continental group and population. In each continental group (Supplemental Figure 3A), the DAF spectrum is characterized by a high proportion of low-frequency SNPs (>60% SNPs in the first bin (DAF 0-0.1)). The first bin also shows that more low-frequency SNPs are found in African than non-African populations. The number of low-frequency polymorphic sites decreases in a step-wise manner from Africans to Europeans to Indians to East Asians (634, 542, 533, and 439, respectively), reflecting an overall decrease in diversity with increasing distance from Africa. East Asians have more intermediate SNPs (DAF $\approx$ 0.4 – 0.7), possibly caused by stronger historical bottlenecks. Within each Indian population, the DAF pattern remains similar (Supplemental Figure 3B). The excess of the rare SNPs is less apparent at the population level, possible due to the small sample size (22-24 individuals) of each population.

Comparison between sequence and SNP microarray data

To quantify the difference between resequencing data and SNP microarray data, we compared the distribution of derived-alleles obtained by resequencing with SNP genotypes obtained by microarray genotyping (Supplemental Figure 4). For the ENCODE sequence data, number of polymorphic sites and the allele frequency distribution were calculated using the HapMap YRI (60), HapMap CEU (60), combined randomly selected HapMap CHB/JPT (60) and South Indians (60; Brahmin, Mala, Madiga, Irula). To obtained comparative data from microarrays, a contiguous set of SNPs on chromosome 12, equal in number to that found by sequencing, was selected randomly from the Affymetrix 250K NspI microarray genotypes [3] for each population (1000 replicates).

For both the sequence and the microarray data, the number of polymorphic sites is higher in Africans than non-Africans. Consistent with ascertainment strategies, however, low-frequency polymorphisms (< 0.2) are significantly under-represented and high-frequency polymorphisms are over-represent in the microarray data for all groups (Supplemental Figure 4). These results demonstrate the necessity of full sequence data sets to accurately assess genetic variation in any major population.

Comparison of three-population and four-population out-of-Africa models for the ∂a∂i analysis

We compared three general three-population models, each with a different set of parameters. The maximum-likelihood values of each model for each of the three-population dataset are shown in the Supplemental Figure 5. The likelihood ratio tests demonstrate that models allowing exponential growth in the two Eurasian continental groups are significantly better than the models with constant population size in both Africa-East Asia-Europe (p=0.004) and Africa-India-Europe (p=0.021) models. Adding migration rate estimates (*ooa_mig*, 11 parameters) among populations does not significantly improve the model fitting (p>0.7) compare to the model without migration (*ooa_simple*, 7 parameters). We then compared three general four-population models, each with a different set of parameters. The maximum-likelihood values for each four-population models are shown in the Supplemental Figure 6. As with the three-population models, models allowing exponential growth in the Eurasian continental groups are significantly more likely than models with constant population size (p<0.01). Among the two models allowing exponential growth, adding migration rate estimates (*ooa_fourpop_growth_mig*, 13 parameters) among groups does not significantly improve the model fitting (p>0.85) compare to the model without migration (*ooa_fourpop_growth*, 9 parameters). Therefore, in the final analysis we estimated the parameters using the three-population *ooa_growth* model and the four-population *ooa_fourpop_growth* model in the interest of minimizing the number of parameters estimated and improving the speed of computation.

∂a∂i analysis at the population level

Because of the limited sample size in individual populations, we performed two-population split-with-migration analysis at the population level (Supplemental Figure 9). The results from the two-population model showed that the pattern observed in the analyses of continental groups remained largely the same (Supplemental Table 4). The CIs around the estimates are generally larger, indicating the loss of power due to the smaller sample sizes of the populations compared to the continental groups. In general, Indian populations have the shortest divergence times from

the HapMap European populations, especially HapMap TSI. With the exception of CHD, there is little migration between Indian populations and HapMap non-Indian populations. It is noteworthy that Eurasian populations in general have a shorter divergence time with HapMap LWK (from East Africa) than HapMap YRI (from West Africa). This result might reflect significant population variation within Africa before the out-of-Africa migration. HapMap GIH diverged from south Indian populations between 1.2 kya (Irula) and 15.3 kya (Mala/Madiga), and there is no substantial estimated migration after the divergence (Supplemental Table 4). We were unable to confidently estimate the population relationship among South Indian populations, probably both due to the lack of power in our dataset, and the closely shared history and high level of migration among these populations.

References

1. Ramensky V, Bork P, Sunyaev S: **Human non-synonymous SNPs: server and survey**. *Nucleic Acids Res* 2002, **30**(17):3894-3900.
2. Kumari U, Tan EK: **LRRK2 in Parkinson's disease: genetic and clinical studies from patients**. *FEBS J* 2009, **276**(22):6455-6463.
3. Xing J, Watkins WS, Witherspoon DJ, Zhang Y, Guthery SL, Thara R, Mowry BJ, Bulayeva K, Weiss RB, Jorde LB: **Fine-scaled human genetic structure revealed by SNP microarrays**. *Genome Res* 2009, **19**(5):815-825.
4. Xing J, Watkins WS, Shlien A, Walker E, Huff CD, Witherspoon DJ, Zhang Y, Simonson TS, Weiss RB, Schiffman JD, Malkin D, Woodward SR, Jorde LB: **Toward a more uniform sampling of human genetic diversity: A survey of worldwide populations by high-density genotyping**. *Genomics* 2010, **96**(4):199-210.

Supplemental Tables

Supplemental Table 1: Validation rate of Indian-specific SNPs

Minor-allele Count	*Experiments**	*Validated*	*Validation Rate (%)*	*Loci**	*Validated*	*Validation Rate (%)*
1	67	57	85.1	67	57	85.1
2-5	78	54	69.2	38	23	60.5
> 5	64	54	84.4	14	12	85.7
Total	209	165	79.6	119	92	77.1

* One validation experiment represents the genotyping of one SNP in one individual. One validation locus represents the genotyping of one SNP locus in all individuals included in the validation process.

Supplemental Table 2: Pairwise F_{ST} values (%) between Indian populations

	Brahmin	*GIH*	*Mala/Madiga*	*Yadava*	*Irula*
Brahmin	-				
GIH	9.3	-			
Mala/Madiga	0.9	7.2	-		
Yadava	3.4	8.4	0.1	-	
Irula	8.5	10.4	3.3	2.1	-

Supplemental Table 3: Genetic diversity in continental groups and populations after removing unconfirmed genotypes in Indian samples.

	nInd	*S*	*Sp*	*θ*	*π (x10^{-5})*	*H (x10^{-5})*	*Tajima's D*	*p*
**Continent**								
India	152	514	218	81.68 (79.77-83.60)	83.38 (78.90-87.85)	77.3	0.06	0.95
Africa	152	656	416	104.25 (101.82-106.68)	85.28 (80.71-89.86)	78.03	-0.57	0.57
Europe	152	535	205	85.02 (83.03-87.01)	74.64 (70.63-78.65)	67.95	-0.38	0.7
East Asia	152	436	186	69.29 (67.66-70.92)	73.61 (69.66-77.57)	73.1	0.19	0.85
**Population**								
Brahmin	23	285	15	64.85 (59.30-70.39)	74.99 (64.43-85.54)	59.86	0.57	0.57
GIH	24	282	47	63.54 (58.27-68.81)	72.41 (62.45-82.38)	60.96	0.51	0.61
Irula	23	285	16	64.85 (59.30-70.39)	82.22 (70.66-93.78)	94.7	0.98	0.33
Mala/Madiga	24	328	40	73.91 (67.79-80.02)	83.65 (72.15-95.15)	88.56	0.48	0.63
Yadava	22	310	29	71.26 (64.98-77.54)	88.47 (75.74-101.19)	92.54	0.89	0.37
LWK	24	359	85	80.89 (74.21-87.57)	82.51 (71.17-93.86)	85.81	0.07	0.94
YRI	24	349	91	78.64 (72.14-85.14)	82.03 (70.75-93.31)	76.86	0.16	0.87
CEU	24	262	43	59.04 (54.13-63.94)	70.64 (60.91-80.37)	77.67	0.72	0.47
TSI	24	298	58	67.15 (61.58-72.71)	73.95 (63.78-84.13)	72.54	0.37	0.71
CHB	24	254	34	57.23 (52.47-61.99)	76.49 (65.97-87.01)	78.88	1.22	0.22
CHD	24	212	24	47.77 (43.78-51.76)	69.87 (60.24-79.49)	72.34	1.68	0.09
JPT	24	236	34	53.18 (48.75-57.61)	73.66 (63.52-83.80)	62.88	1.4	0.16

nInd: number of individuals; *S*: number of segregating sites; *Sp*: number of private segregating sites; *θ*: estimated theta ($4N_eu$) from *S*; *π*: Nucleotide diversity; *H*: observed heterozygosity; *Tajima's D*: Tajima's *D*; *p*: p value for Tajima's D test. Confidence intervals of *θ* and *π* are shown in parenthesis.

Supplemental Table 4: *∂a∂i* inferred parameters for divergence between Indian and HapMap populations.

Pop1	*Pop2*	N_A	N_1	N_2	*T (kya)*	$m_{1->2}$ *(x10^{-5})*	$m_{2->1}$ *(x10^{-5})*
Brahmin	GIH	13591	2586 (93-5164)	2717 (114-5110)	9.2 (0.3-17.7)	0.00 (0.00-1.06)	0.00 (0.00-3.95)
Irula	GIH	14778	270 (14-2133)	276 (15-2245)	1.2 (0.1-10.1)	0.20 (0.00-2.05)	0.31 (0.00-2.42)
Mala_Madiga	GIH	14548	6516 (2703-10735)	3326 (1351-5325)	15.3 (5.8-26.1)	0.00 (0.00-0.58)	0.00 (0.00-4.09)
Yadava	GIH	14781	1981 (0-0)	1362 (0-0)	6.3 (0.0-0.0)	0.01 (0.00-0.00)	0.00 (0.00-0.00)
Brahmin	TSI	13934	3864 (1690-6151)	4066 (1754-6610)	17.6 (7.1-28.4)	0.00 (0.00-4.68)	0.00 (0.00-3.46)
GIH	TSI	13170	5531 (2921-8359)	5701 (3126-9108)	18.8 (9.5-27.5)	0.00 (0.00-2.35)	0.01 (0.00-0.71)
Irula	TSI	15005	2585 (782-4300)	2833 (910-4935)	14.9 (4.0-25.6)	0.00 (0.00-0.84)	0.00 (0.00-1.27)
Mala_Madiga	TSI	14407	9608 (6145-13751)	5754 (3740-8882)	27.6 (16.9-40.2)	0.00 (0.00-0.07)	0.02 (0.00-2.52)
Yadava	TSI	15015	4814 (2533-7233)	3804 (1969-5788)	22.1 (11.0-33.3)	0.00 (0.00-2.65)	0.00 (0.00-4.43)
Brahmin	CEU	13588	4707 (2643-6856)	5591 (3143-8468)	22.1 (11.9-33.0)	0.00 (0.00-0.49)	0.00 (0.00-3.18)
GIH	CEU	12871	7082 (4929-9844)	8544 (5999-12035)	31.2 (21.1-42.2)	0.00 (0.00-0.02)	0.00 (0.00-0.01)
Irula	CEU	14681	3245 (1621-5049)	3729 (1787-5992)	20.0 (9.4-32.1)	0.00 (0.00-5.26)	0.00 (0.00-1.69)
Mala_Madiga	CEU	13933	10925 (7396-15819)	6895 (4577-9887)	31.7 (21.1-44.5)	0.00 (0.00-1.65)	0.00 (0.00-1.57)
Yadava	CEU	14690	5531 (3264-8088)	4601 (2758-6727)	26.6 (15.4-38.9)	0.00 (0.00-3.44)	0.00 (0.00-3.21)
Brahmin	CHB	13889	5832 (4183-7581)	4763 (3258-7040)	57.3 (35.6-129.7)	0.00 (0.00-0.00)	7.23 (0.00-20.10)
GIH	CHB	13271	7002 (4940-9330)	4573 (3335-6865)	40.5 (28.4-154.9)	0.00 (0.00-0.00)	0.00 (0.00-28.70)
Irula	CHB	14599	4031 (2211-6244)	2535 (1272-3956)	21.8 (10.7-35.8)	0.00 (0.00-0.00)	0.00 (0.00-0.01)
Mala_Madiga	CHB	14130	11279 (7747-15709)	4136 (2927-5900)	42.6 (29.1-72.3)	0.00 (0.00-0.00)	0.00 (0.00-26.07)
Yadava	CHB	16185	5337 (3884-7964)	6040 (2042-6595)	141.4 (22.3-182.2)	0.00 (0.00-0.00)	27.57 (0.00-31.01)
Brahmin	CHD	13681	4518 (2488-6515)	2926 (1217-5263)	32.9 (11.2-90.5)	0.00 (0.00-0.73)	22.52 (0.00-37.34)
GIH	CHD	12408	6640 (2608-9308)	4533 (1017-5744)	49.0 (6.1-78.8)	0.00 (0.00-0.59)	39.59 (1.59-44.29)
Irula	CHD	14087	1266 (27-4231)	590 (14-1998)	4.4 (0.1-18.1)	0.00 (0.00-1.34)	21.16 (0.00-37.19)

Mala_Madiga	CHD	14093	11168 (5554-15886)	4016 (1275-5887)	65.1 (10.8-124.8)	0.00 (0.00-1.60)	35.47 (0.00-38.74)
Yadava	CHD	14346	7974 (1053-8479)	3762 (394-5250)	68.9 (3.3-114.7)	0.00 (0.00-1.24)	34.84 (0.62-38.11)
Brahmin	JPT	13834	6218 (4640-7972)	4448 (3156-6496)	67.5 (43.0-157.9)	0.00 (0.00-0.00)	8.09 (0.00-20.23)
GIH	JPT	14039	5512 (4728-9551)	6028 (2909-6833)	134.0 (28.0-182.3)	0.00 (0.00-0.00)	29.62 (0.00-36.14)
Irula	JPT	14330	5035 (3122-7366)	2594 (1556-3970)	27.2 (15.4-48.9)	0.00 (0.00-0.00)	0.00 (0.00-29.13)
Mala_Madiga	JPT	13913	12390 (7985-16762)	3803 (2856-5903)	45.7 (33.4-173.5)	0.00 (0.00-0.00)	0.00 (0.00-31.84)
Yadava	JPT	16402	5713 (4620-8840)	5400 (2097-6445)	145.1 (25.7-198.7)	0.00 (0.00-0.01)	26.72 (0.00-31.92)
Brahmin	LWK	14438	7991 (6259-10628)	13052 (10040-16186)	79.1 (62.0-121.6)	0.00 (0.00-5.93)	0.00 (0.00-0.00)
GIH	LWK	13794	9078 (7142-11151)	15426 (12362-19498)	80.1 (63.3-100.8)	0.00 (0.00-1.19)	0.00 (0.00-0.00)
Irula	LWK	15667	6304 (4719-8002)	11054 (8177-14832)	65.7 (47.4-85.1)	0.00 (0.00-0.00)	0.00 (0.00-0.00)
Mala_Madiga	LWK	14462	13001 (10199-16355)	14335 (11208-17641)	86.7 (67.6-112.7)	0.00 (0.00-1.27)	0.00 (0.00-0.00)
Yadava	LWK	15088	9300 (7322-11617)	12501 (9657-16239)	81.8 (63.4-101.5)	0.00 (0.00-0.01)	0.00 (0.00-0.00)
Brahmin	YRI	13782	9374 (7206-11792)	11123 (8835-13595)	108.4 (81.9-152.5)	1.16 (0.00-5.00)	0.00 (0.00-0.00)
GIH	YRI	13360	9849 (8095-11864)	12887 (10394-15706)	103.7 (83.2-129.8)	0.00 (0.00-1.03)	0.00 (0.00-0.00)
Irula	YRI	15420	7100 (5586-8743)	9623 (7399-12187)	83.8 (64.2-105.2)	0.00 (0.00-0.00)	0.00 (0.00-0.00)
Mala_Madiga	YRI	14150	13231 (10636-16175)	12120 (9718-14580)	108.0 (88.3-138.7)	0.00 (0.00-2.14)	0.00 (0.00-0.00)
Yadava	YRI	14946	9711 (7870-11664)	10628 (8496-13224)	100.7 (80.4-123.8)	0.00 (0.00-0.58)	0.00 (0.00-0.00)

* Confidence intervals are shown in parentheses.

Supplemental Figures

Supplemental Figure 1: Principal components analysis of all populations. The first two PCs are shown. The percentage of variance explained by each PC is shown on the axis. Each population is represented by one dot.

Supplemental Figure 2: Individual relationship in the normalized dataset. A) Principal components analysis. PCA was performed on pairwise allele-sharing distance between each pair of individuals as previously described [4]. The first two PCs are shown. The percentage of variance explained by each PC is shown on the axis. Each individual is represented by one dot. **B) Individual grouping inferred by *ADMIXTURE*.** Results from K=2 to K=4 are shown. Each individual's genome is represented by a vertical bar composed of colored sections, where each section represents the proportion of an individual's ancestry derived from one of the K ancestral populations. Individuals are arrayed horizontally and grouped by continental groups as indicated.

Supplemental Figure 3: DAF distributions of A) four major continental groups and B) south Indian populations. The number of polymorphic SNPs for each population is shown by the DAF bin.

Supplemental Figure 4: DAF distributions of sequencing data and microarray data. The DAF spectra for all polymorphic SNPs in the ENCODE region (blue) and for the Affymetrix 250K NspI microarray SNPs (red) in four major population groups (60 individuals each). Error bars correspond to twice the standard deviation of 1000 resampled replicates.

Supplemental Figure 5: Comparison of three-population out-of-Africa models. The maximum-likelihood estimate for each continental group combination is shown for three models with different parameter sets.

Supplemental Figure 6: Comparison of four-population out-of-Africa models. The maximum-likelihood estimate for each continental group combinations are shown for three models with different parameter sets.

Supplemental Figure 7: Three-population *ooa_growth* model optimization function. The python program used to estimate the parameters using the *ooa_growth* model. Parameters used in the final analysis, including the function calls, grid sizes, initial parameters, and parameter boundaries are shown.

Supplemental Figure 8: Four-population *ooa_fourpop_growth* model optimization function. The python program used to estimate the parameters using the *ooa_fourpop_growth* model. Parameters used in the final analysis, including the function calls, grid sizes, initial parameters, fixed parameters, and parameter boundaries are shown.

Supplemental Figure 9: Two-population *split_mig* model. In this model, two populations split from an ancestral population in the past and maintain constant population sizes until the present, with possible inter-population migrations. The population divergence time (T), effective population sizes of the ancestral population (N_A) and the two current populations (N_1 and N_2, respectively), as well as migration rates between the two populations ($m_{1->2}$ and $m_{2->1}$, respectively) are estimated.

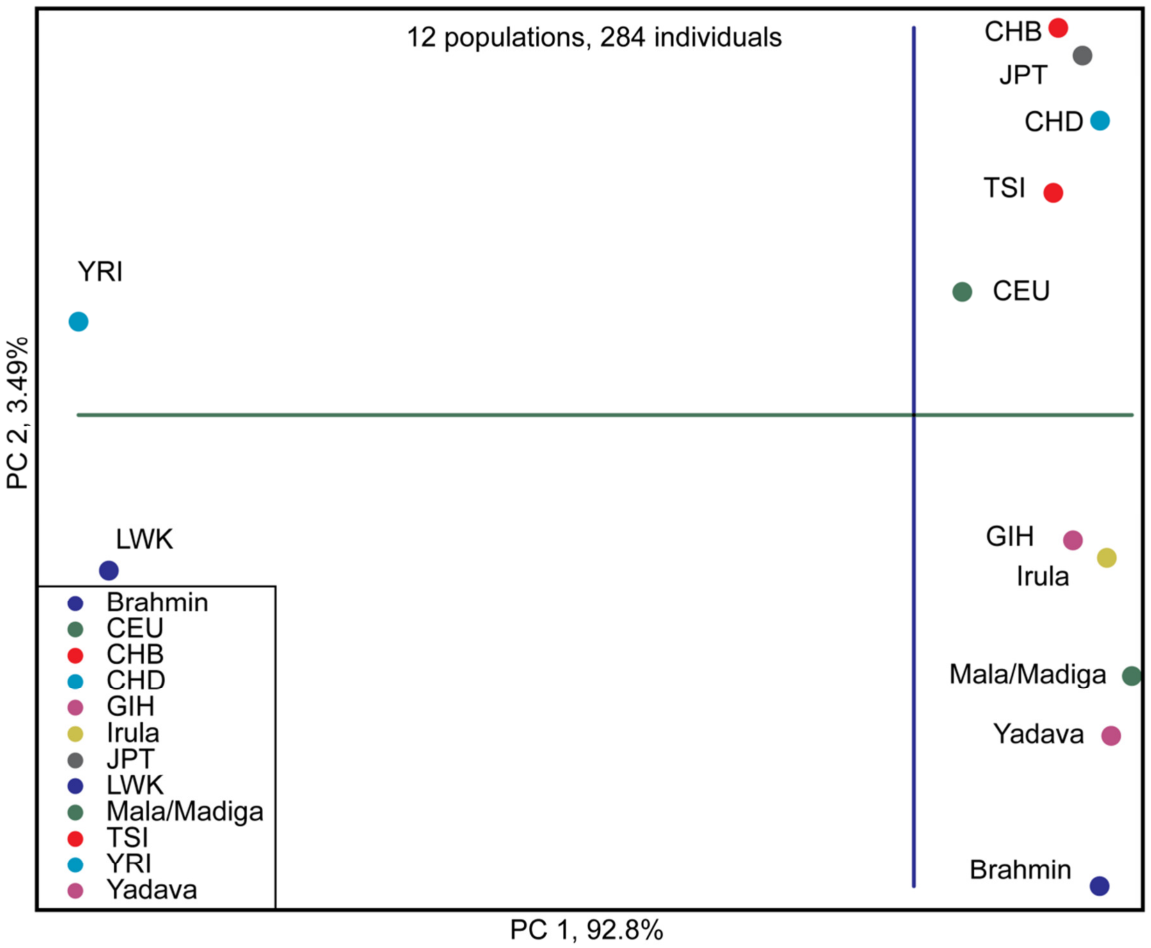

Supplemental Figure 1

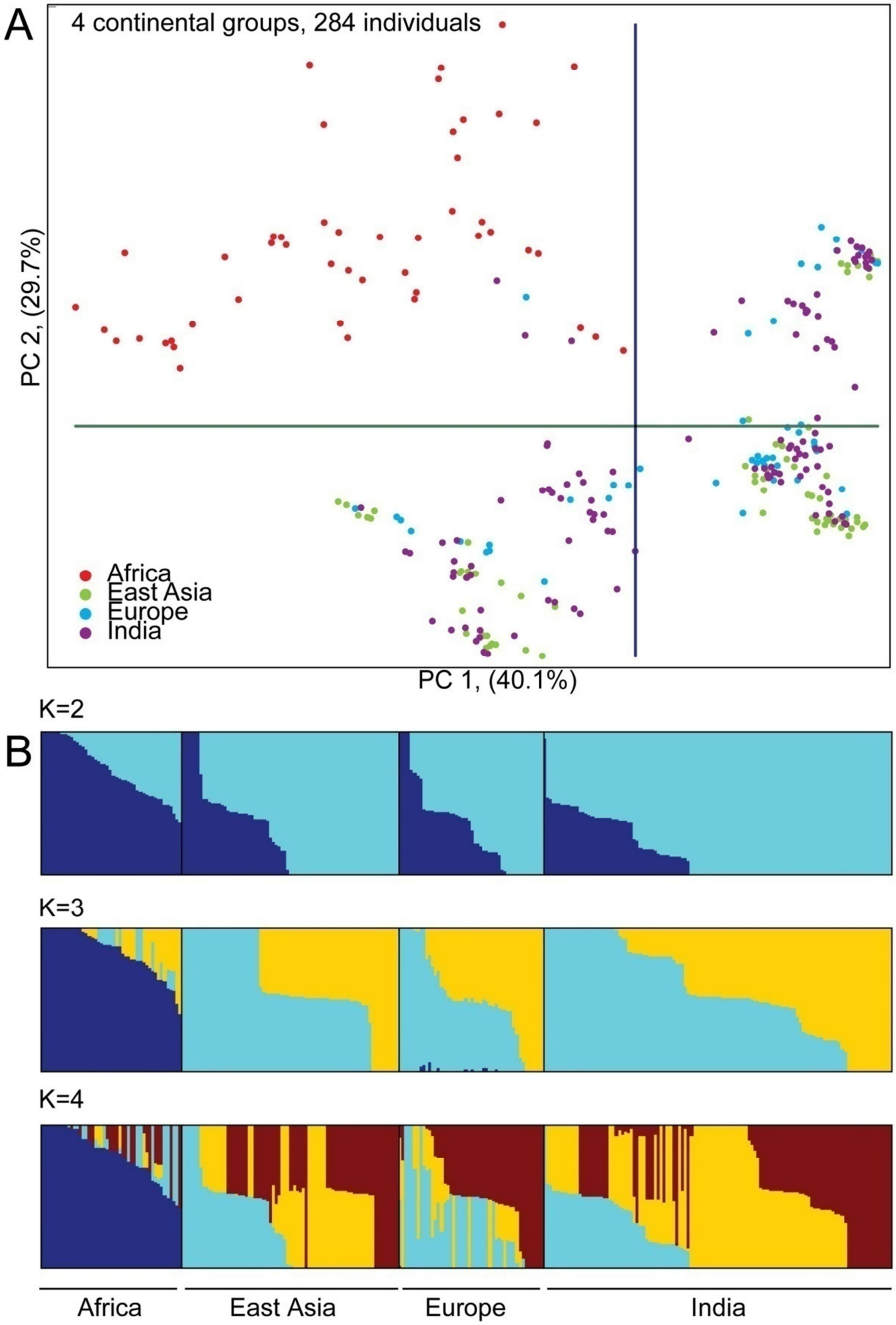

Supplemental Figure 2

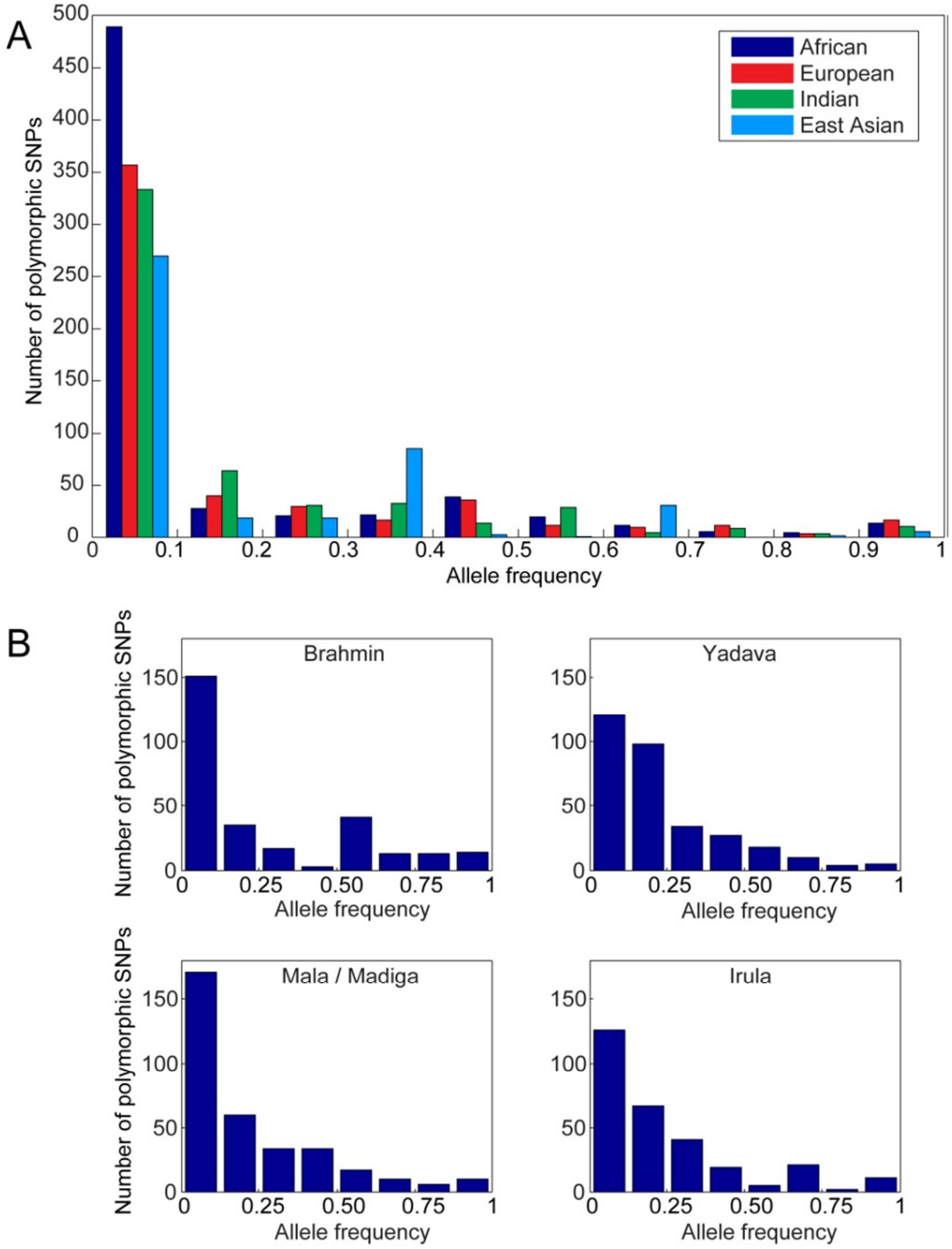

Supplemental Figure 3

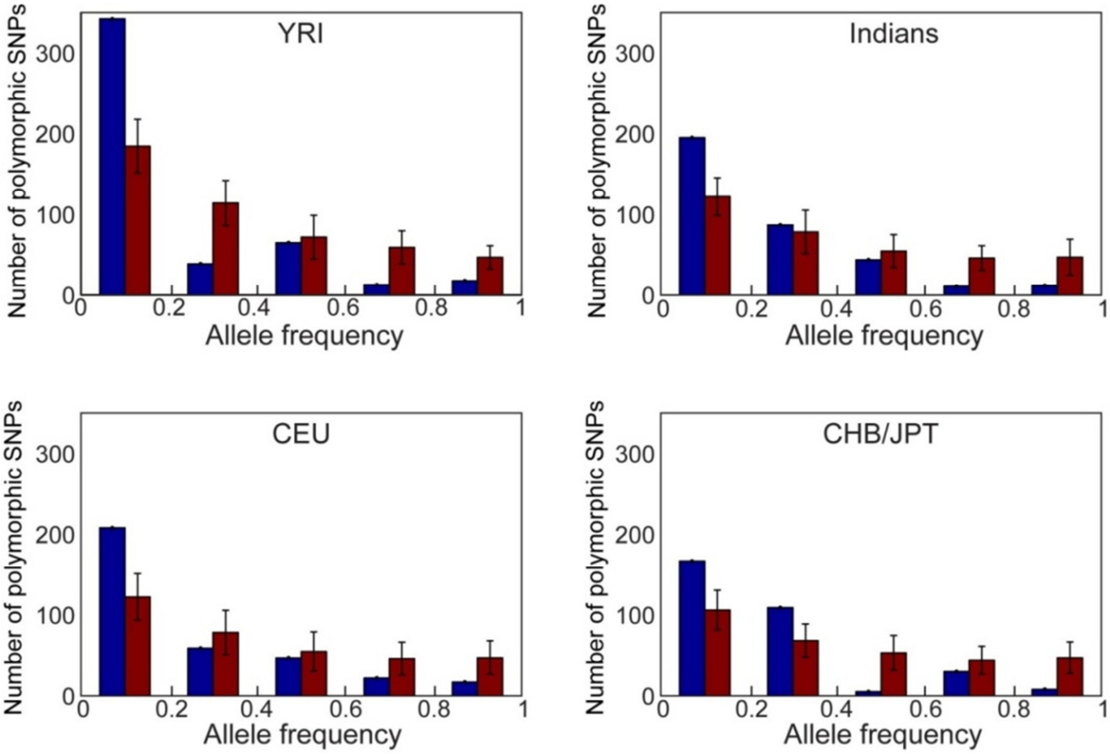

Supplemental Figure 4

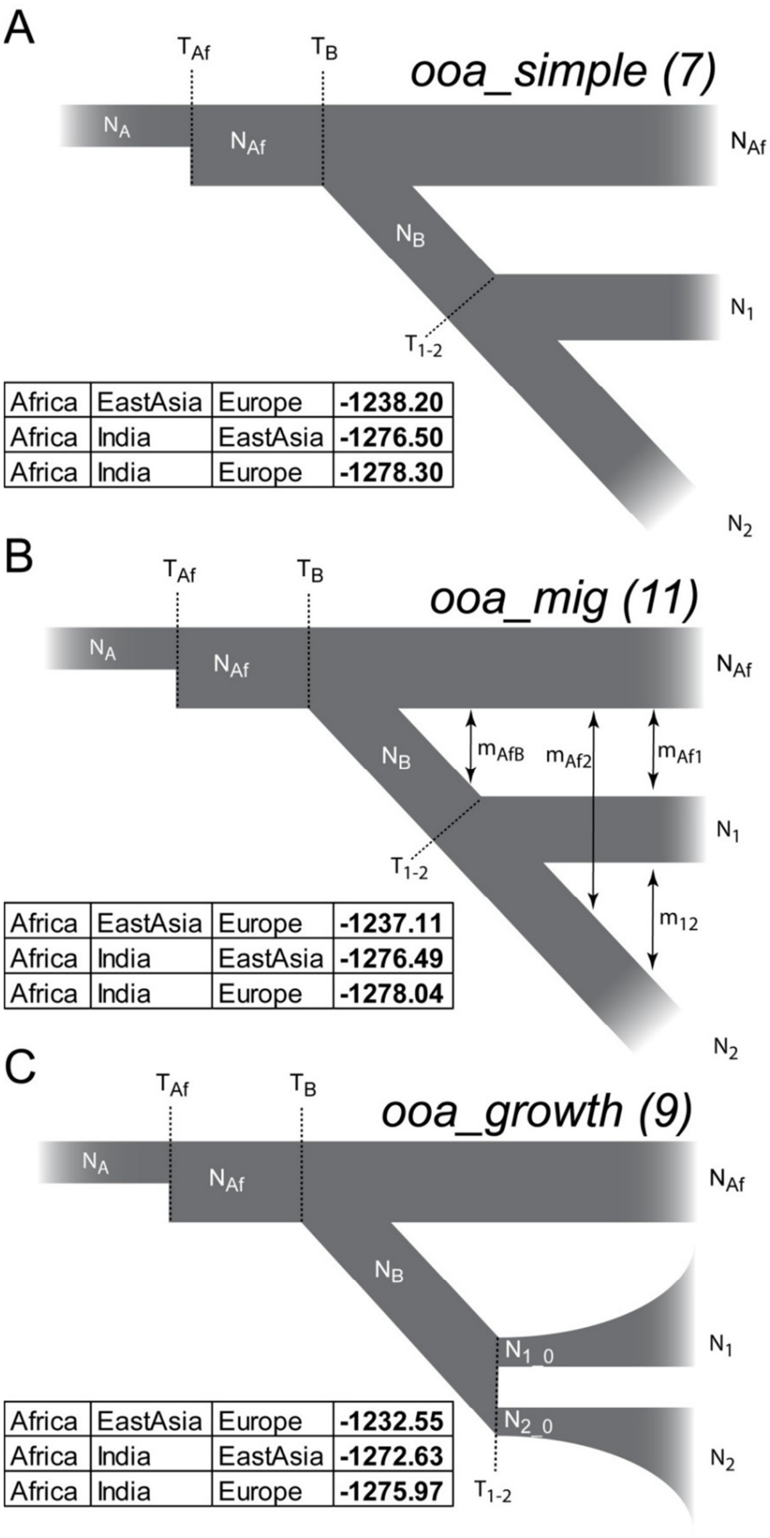

A

Africa	EastAsia	Europe	**-1238.20**
Africa	India	EastAsia	**-1276.50**
Africa	India	Europe	**-1278.30**

B

Africa	EastAsia	Europe	**-1237.11**
Africa	India	EastAsia	**-1276.49**
Africa	India	Europe	**-1278.04**

C

Africa	EastAsia	Europe	**-1232.55**
Africa	India	EastAsia	**-1272.63**
Africa	India	Europe	**-1275.97**

Supplemental Figure 5

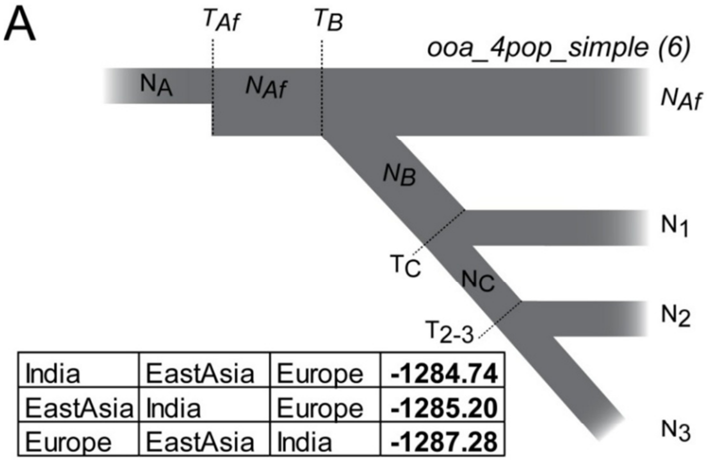

India	EastAsia	Europe	**-1284.74**
EastAsia	India	Europe	**-1285.20**
Europe	EastAsia	India	**-1287.28**

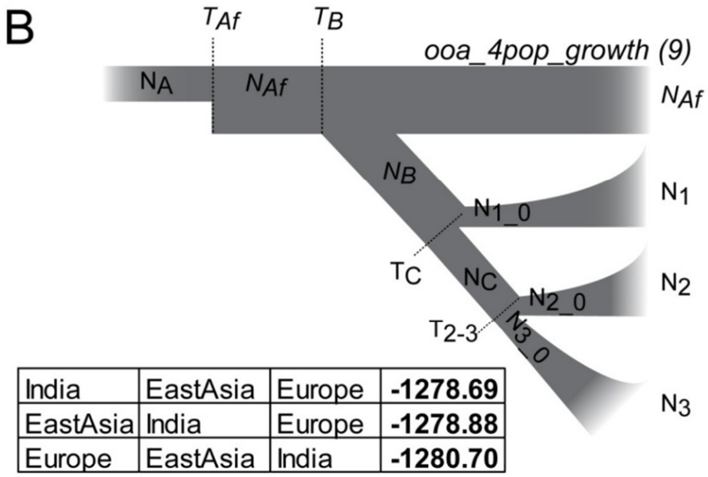

India	EastAsia	Europe	**-1278.69**
EastAsia	India	Europe	**-1278.88**
Europe	EastAsia	India	**-1280.70**

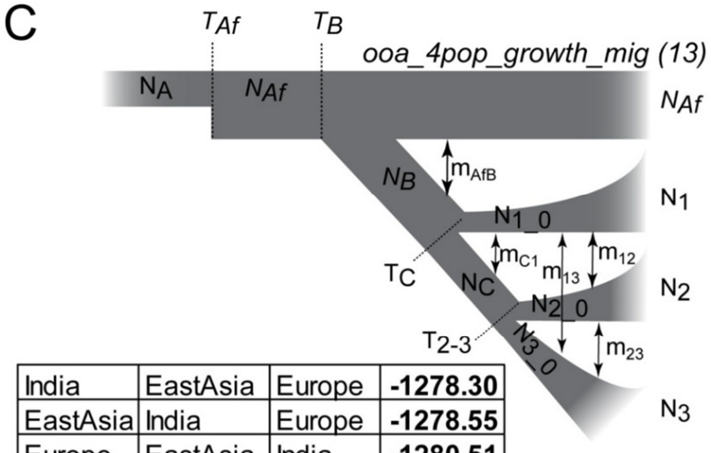

India	EastAsia	Europe	**-1278.30**
EastAsia	India	Europe	**-1278.55**
Europe	EastAsia	India	**-1280.51**

Supplemental Figure 6

```
import dadi
import numpy
import sys
from numpy import array
import custom_pop_models

# Load the data
pop1=sys.argv[1]
pop2=sys.argv[2]
pop3=sys.argv[3]
infile=str(sys.argv[4])

ind_projection=50
dd = dadi.Misc.make_data_dict(infile)
data = dadi.Spectrum.from_data_dict(dd, [pop1,pop2,pop3],
[ind_projection,ind_projection,ind_projection])
ns = data.sample_sizes

# Grid point settings will be used for extrapolation.
pts_l = [70,80,90]

# Use modified split-migration model which allows asymmetry migration rate.
func = custom_pop_models.ooa_growth

# Parameters: nuAf, nuB,nu1_0,  nu1, nu2_0,  nu2, TAf,  TB,T1-2
params = array([ 1,   1,  0.1,    1,   0.1,    1,0.01,0.01,0.01])
upper_bound =  [10,  10,    2,   10,     2,   10,   1,   1,   1]
lower_bound =[1e-3,1e-3, 1e-3, 1e-3,  1e-3, 1e-3,1e-5,1e-5,1e-5]

# Make the extrapolating version of the model function.
func_ex = dadi.Numerics.make_extrap_func(func)

# Perturb our parameter array before optimization.
p0 = dadi.Misc.perturb_params(params, fold=1, lower_bound=lower_bound,
upper_bound=upper_bound)

# Perform optimization.
popt = dadi.Inference.optimize_log(p0, data, func_ex,
                                   pts_l,
                                   lower_bound=lower_bound,
                                   upper_bound=upper_bound,
                                   verbose=len(params))

# The optimal value of theta given the model.
model = func_ex(popt, ns, pts_l)
theta = dadi.Inference.optimal_sfs_scaling(model, data)

# The optimal value of log-likelihood given the model.
ll_opt = dadi.Inference.ll_multinom(model, data)

# Print theta along with optimized parameters
print 'Optimized parameters', repr([theta,ll_opt,popt])
```

Supplemental Figure 7

```
import dadi
import numpy
import sys
from numpy import array
import custom_pop_models

# Load the data
pop1=sys.argv[1]
pop2=sys.argv[2]
pop3=sys.argv[3]
infile=str(sys.argv[4])

ind_projection=50
dd = dadi.Misc.make_data_dict(infile)
data = dadi.Spectrum.from_data_dict(dd, [pop1,pop2,pop3], [ind_projection,ind_projection,ind_projection])
ns = data.sample_sizes

# Grid point settings will be used for extrapolation.
pts_l = [70,80,90]

# Use modified split-migration model which allows asymmetry migration rate.
func = custom_pop_models.ooa_fourpop_growth

# Parameters: nuAf, nuB, nuC,nu1_0, nu1, nu2_0, nu2,  nu3_0,  nu3,  TAf,  TB,  TC, T2-3
params = array([ 1,   1,   1,  0.1,    1,  0.1,    1,   0.1,    1, 0.01,0.01,0.01,0.01])
upper_bound =  [10,  10,  10,    2,   10,    2,   10,     2,   10,    1,   1,   1,   1]
lower_bound =[1e-3,1e-3,1e-3, 1e-3, 1e-3, 1e-3, 1e-3,  1e-3, 1e-3, 1e-5,1e-5,1e-5,1e-5]

# Fixed Parameters: nuAf, nuB, TAf, TB
nuAf=1.4417
nuB=0.91561
TAf=0.04149
TB=0.073183

params_fix = array([nuAf, nuB, None, None, None, None, None, None, None, TAf, TB, None, None])

# Make the extrapolating version of the model function.
func_ex = dadi.Numerics.make_extrap_func(func)
```

```
# Perturb our parameter array before optimization.
p0 = dadi.Misc.perturb_params(params, fold=1, lower_bound=lower_bound, upper_bound=upper_bound)

# perform optimization
popt = dadi.Inference.optimize_log(p0, data, func_ex,
                                   pts_l,
                                   lower_bound=lower_bound,
                                   upper_bound=upper_bound,
                                   verbose=len(params),
                                   fixed_params = params_fix)

# The optimal value of theta given the model.
model = func_ex(popt, ns, pts_l)
theta = dadi.Inference.optimal_sfs_scaling(model, data)

# The optimal value of log-likelihood given the model.
ll_opt = dadi.Inference.ll_multinom(model, data)

# Print theta along with optimized parameters
print 'Optimized parameters', repr([theta,ll_opt,popt])
```

Supplemental Figure 8

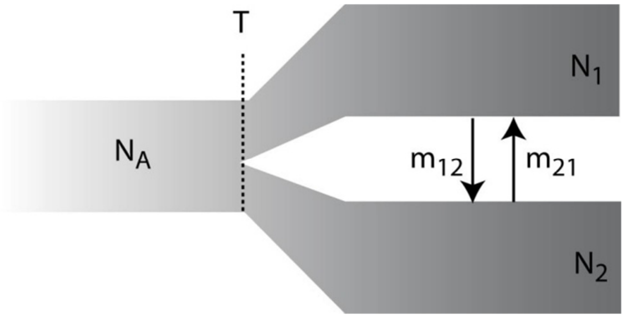

Supplemental Figure 9
